# Nitric Oxide Donor Sodium Nitroprusside Reduces Racemic Ketamine—But Not Esketamine-Induced Pain Relief

**DOI:** 10.1021/acsptsci.4c00133

**Published:** 2024-06-21

**Authors:** Albert Dahan, Simone Jansen, Rutger van der Schrier, Elise Sarton, David Dadiomov, Monique van Velzen, Erik Olofsen, Marieke Niesters

**Affiliations:** † Department of Anesthesiology, Leiden University Medical Center, 2333 ZA Leiden, The Netherlands; ‡ PainLess Foundation, 2333 ZA Leiden, The Netherlands; § USC Alfred E. Mann School of Pharmacy and Pharmaceutical Sciences, Titus Family Department of Clinical Pharmacy, University of Southern California, Los Angeles, California 90089, United States; ∥ Outcomes Research Consortium, Cleveland, Ohio 90089, United States

**Keywords:** ketamine, S-ketamine, R-ketamine, analgesia, nitric oxide, modeling

## Abstract

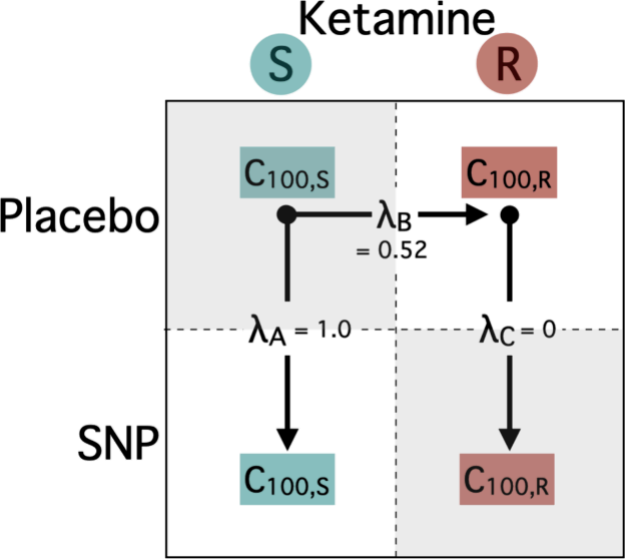

The anesthetic, analgesic and antidepressant drug ketamine produces dissociation with symptoms of psychosis and anxiety, an effect attributed to neuronal nitric oxide depletion following *N*-methyl-d-aspartate blockade. There is evidence that dissociation induced by racemic ketamine, containing both ketamine enantiomers (S- and R-ketamine) but not esketamine (the S-isomer) is inhibited by nitric oxide (NO) donor sodium nitroprusside (SNP). We tested whether a similar intervention would reduce racemic and esketamine-induced analgesia in a randomized double-blind placebo-controlled trial. Seventeen healthy volunteers were treated with 0.5 μg.kg^–1^.min^–1^ SNP or placebo during a 3-h infusion of escalating doses of racemic ketamine (total dose 140 mg) or esketamine (70 mg). Pain pressure threshold (PPT) and arterial blood samples for measurement of S- and R-ketamine and their metabolites, S- and R-norketamine, were obtained. The data were analyzed with a population pharmacokinetic-pharmacodynamic model that incorporated the measured S- and R- ketamine and S- and R-norketamine isomers as input and PPT as output to the model. The potency of the 2 formulations in increasing PPT from baseline by 100% was 0.47 ± 0.12 (median ± standard error of the estimate) nmol/mL for esketamine and 0.62 ± 0.19 nmol/mL for racemic ketamine, reflecting the 52 ± 27% lower analgesic potency of R-ketamine versus S-ketamine. Modeling showed that SNP had no effect on S-ketamine potency but abolished the R-ketamine analgesic effect. Similar observations were made for S- and R-norketamine. Since SNP had no effect on S-ketamine analgesia, we conclude that SNP interacts on R-ketamine nociceptive pathways, possibly similar to its effects on R-ketamine activated dissociation pathways.

The *N*-methyl-d-aspartate (NMDA) receptor antagonist ketamine produces a state of dissociative anesthesia at high dose and pain relief and mood improvement at subanesthetic doses.^1−3^ Dissociative symptoms already occur at low ketamine doses, an effect that usually wanes when plasma concentrations drop but sometimes persist beyond the treatment period.^4^ We recently showed that the nitric oxide (NO) donor, sodium nitroprusside (SNP), reduced dissociative symptoms during the administration of racemic ketamine (which contains isomers R- and S-ketamine) but not during infusion of esketamine (which exclusively contains the S-isomer, S-ketamine).^5^ These data suggest that neuronal nitric oxide depletion due to blockade of the NMDA receptor plays a major role in ketamine activated dissociative pathways within the brain; particularly the pathways activated by R-ketamine are sensitive to NO. Animal data indicate that upon binding of ketamine to the phencyclidine site of the NMDA receptor, neuronal inflow of Ca^2+^-ions is blocked.^6^ This impairs the glutamate-Ca^2^-calmodulin-NO synthase-NO-cyclic guanosine monophosphate (cGMP)-protein kinase pathway (glutamate-cGMP pathway), which is crucial for NO-dependent neuroplasticity, neurotrophic effects and neuroprotection.^7−9^ NMDA receptor hypofunction and the resultant NO depletion is thought to be one of the mechanisms involved in generating ketamine-induced dissociative symptoms that include psychedelic symptoms (hallucinations, paranoia), derealization and depersonalization often with anxiety,^5−9^ similar to symptoms observed in schizophrenia.^10,11^ Several rodent studies (but certainly not all) examining the effect of NO on racemic ketamine- or phencyclidine-induced dissociation, demonstrate that NO derived from SNP reduces psychotic symptoms, memory defects, social withdrawal and anxiety.^12,13^ Interestingly, SNP has been shown to improve psychotic symptoms in patients diagnosed with schizophrenia.^10,14^

There is some evidence that reduction of the dissociative symptoms induced by ketamine reduces therapeutic benefit. For example, a randomized controlled trial showed that masking conscious dissociative ketamine symptoms by treating patients during general anesthesia for elective surgery with ketamine resulted in postoperative antidepressant effects no greater than placebo.^15^ It remains unknown whether the connection between conscious dissociation and ketamine’s therapeutic effects extends beyond its antidepressant properties. Ketamine is extensively used in acute and chronic pain management.^2^ Hence, it is of interest, both from clinical and mechanistic viewpoints, to determine whether NO influences pain relief induced by ketamine, particularly by racemic ketamine. In order to shed some light on this matter, we reanalyzed a data set to evaluate the effect of SNP on esketamine and racemic ketamine-induced dissociation, obtained from healthy volunteers.^5^ Apart from measuring dissociative symptoms, we also collected analgesic responses in that study, and demonstrated earlier that the two end points are correlated.^16^ We here present a preplanned population pharmacokinetic-pharmacodynamic (PKPD) analysis of the influence of SNP on racemic- and esketamine-induced analgesia by measuring the pain pressure threshold during and following a 3-h ketamine infusion. We measured S- and R-ketamine concentrations together with their norketamine metabolites in plasma and constructed a PKPD model of the separate S-ketamine, R-ketamine and norketamine effects on nociception. Our null hypothesis is that SNP is without effect on analgesia induced by both ketamine isomers. We here use the terms esketamine and racemic ketamine for the ketamine formulations, and S-ketamine and R-ketamine for the ketamine enantiomers.

## Results and Discussion

We conducted a population pharmacokinetic-pharmacodynamic model analysis of the impact of NO donor SNP on analgesia induced by two ketamine formulations, racemic ketamine and esketamine in 17 healthy volunteers. S- and R-ketamine, along with their metabolites S- and R-norketamine were incorporated into the pharmacokinetic and pharmacodynamic models. We demonstrate that SNP reduced racemic ketamine-induced analgesic responses by virtually abolishing the R-ketamine contribution of the racemate to effect. Conversely, SNP did not alter esketamine-induced pain relief, indicating that NO does not play a significant role in S-ketamine-mediated nociceptive pathways.

**Figure 1:**
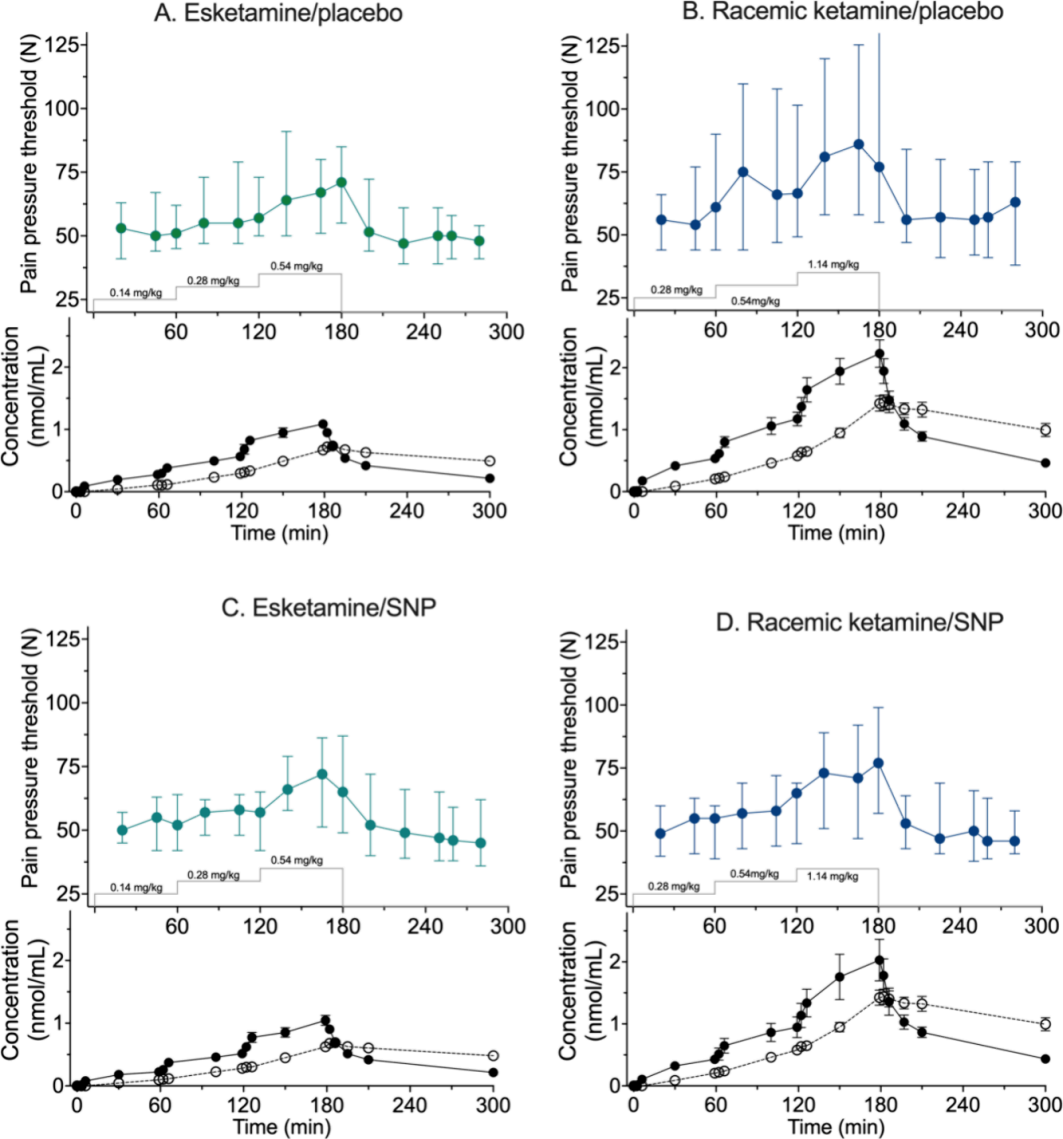
Analgesic responses and plasma concentrations of total ketamine (closed symbols) and total norketamine (open circles) following administration of esketamine and placebo (A), racemic ketamine and placebo (B), esketamine and sodium nitroprusside (SNP) (C), and racemic ketamine and SNP (D). Analgesic data are median ± interquartile range; plasma concentrations are mean ±95% confidence interval. The gray line depicts the esketamine or racemic ketamine dosing strategy.

To get an indication of the drug effect (esketamine and racemic-ketamine) in studies without and with infusion of SNP, we plotted the pain pressure threshold medians ± interquartile ranges in Figure 1 A-D. In agreement with the differences in potency of S-ketamine (IC_50_ 0.8 μM)^17^ and R-ketamine (IC_50_ 1.5 μM),^17^ peak effects following the infusion of esketamine and racemic ketamine differed with peak effect racemic ketamine 86 N versus esketamine 70 N, in studies without SNP infusion. During SNP infusion these values were racemic ketamine peak effect 70 N versus esketamine 72 N. The variability in the data was appreciable, particularly in the baseline data.

**Figure 2:**
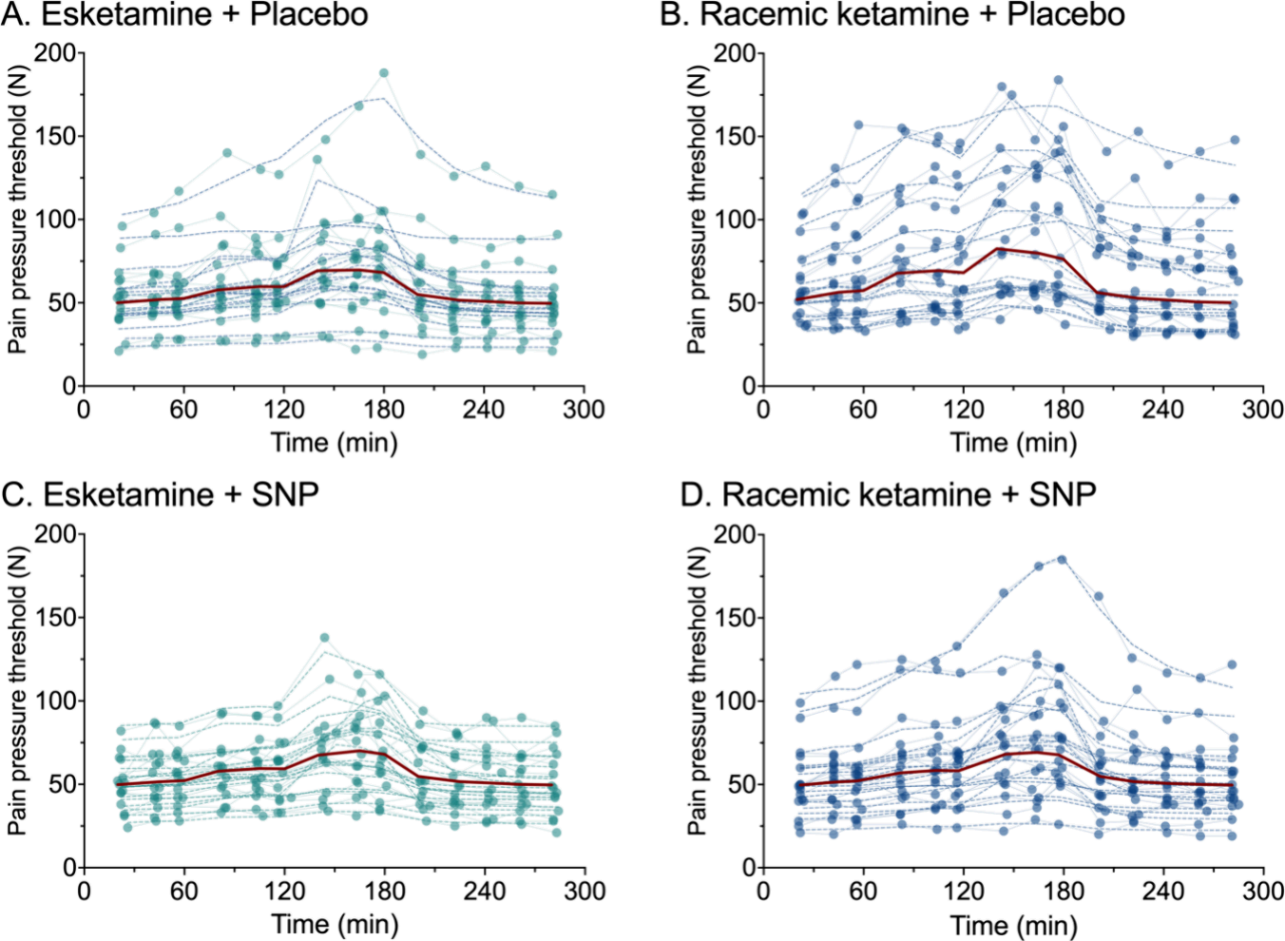
Individual measured pressure pain data (closed symbols) and data fits (broken lines through the data) for the four treatment conditions: esketamine and placebo (A), racemic ketamine and placebo (B), esketamine and sodium nitroprusside (SNP) (C), and racemic ketamine and SNP (D). The population predicted pharmacodynamic response is given by the red lines.

**Figure 3:**
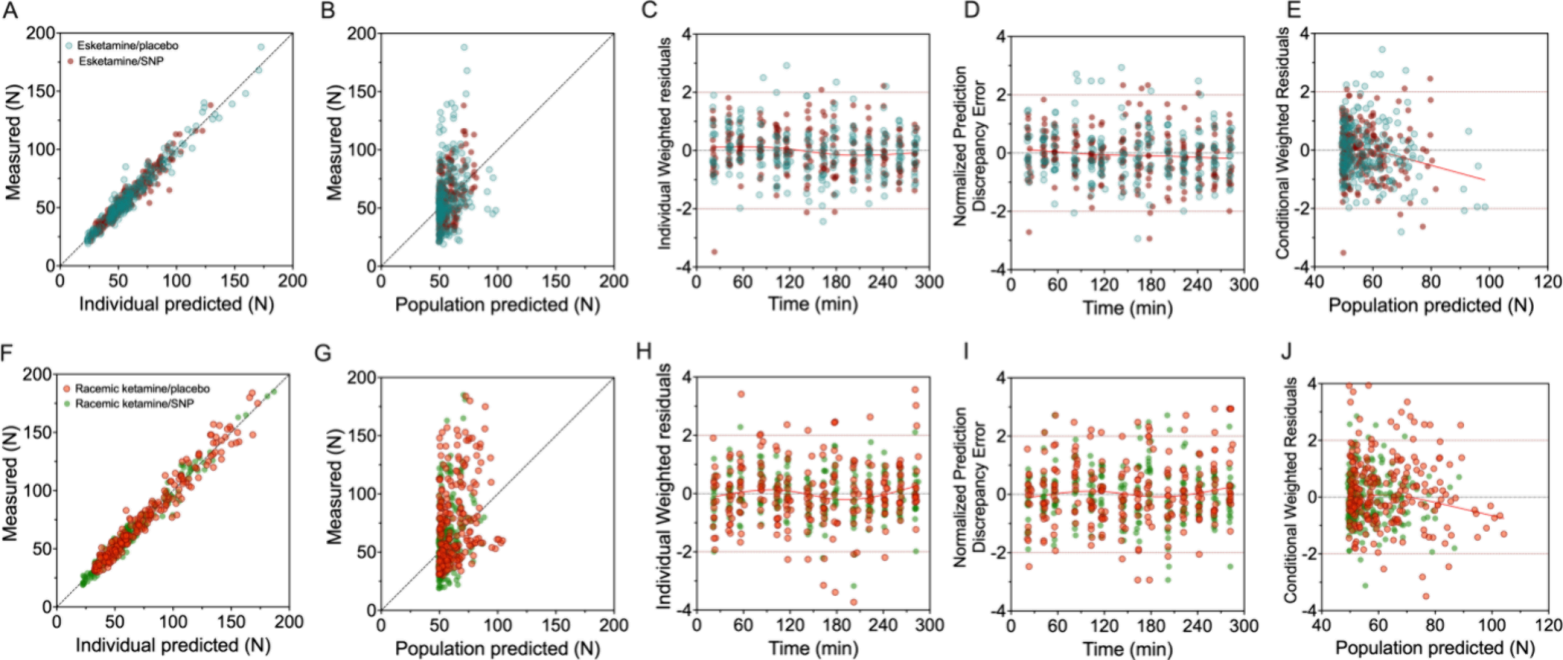
Goodness-of-fit plots for the population pharmacodynamic model following esketamine infusion (panels A–E) and racemic ketamine infusion (panels F–J). Placebo and sodium nitroprusside (SNP) treatment are depicted by different symbol colors. (A and F) Observed pain pressure threshold data versus individual predicted. (B and G) Observed pain pressure threshold versus population predicted. (C and H) Individual weighted residual versus time. (D and I) Normalized prediction discrepancy error versus time. (E and J) Conditioned weighted residuals versus population predicted. Through the data of panels C–E and H–J a spline is fitted to guide the eye (red line).

**Figure 4:**
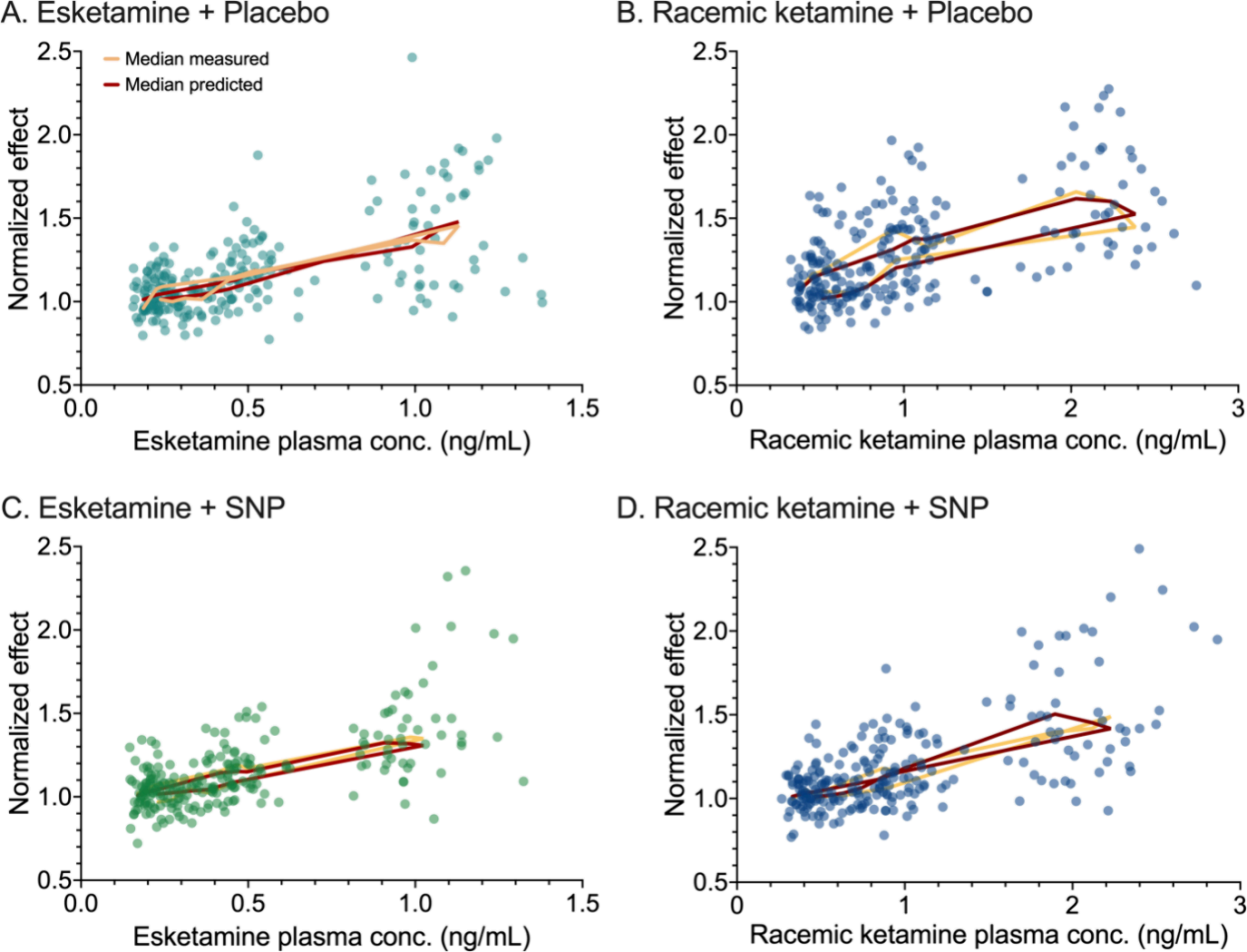
Normalized effect of the raw data (symbols), median measured data (yellow lines), and median predicted data population fits (red lines) versus plasma concentrations of esketamine (panels A and C) and racemic ketamine (panels B and D) without and with sodium nitroprusside.

**Figure 5:**
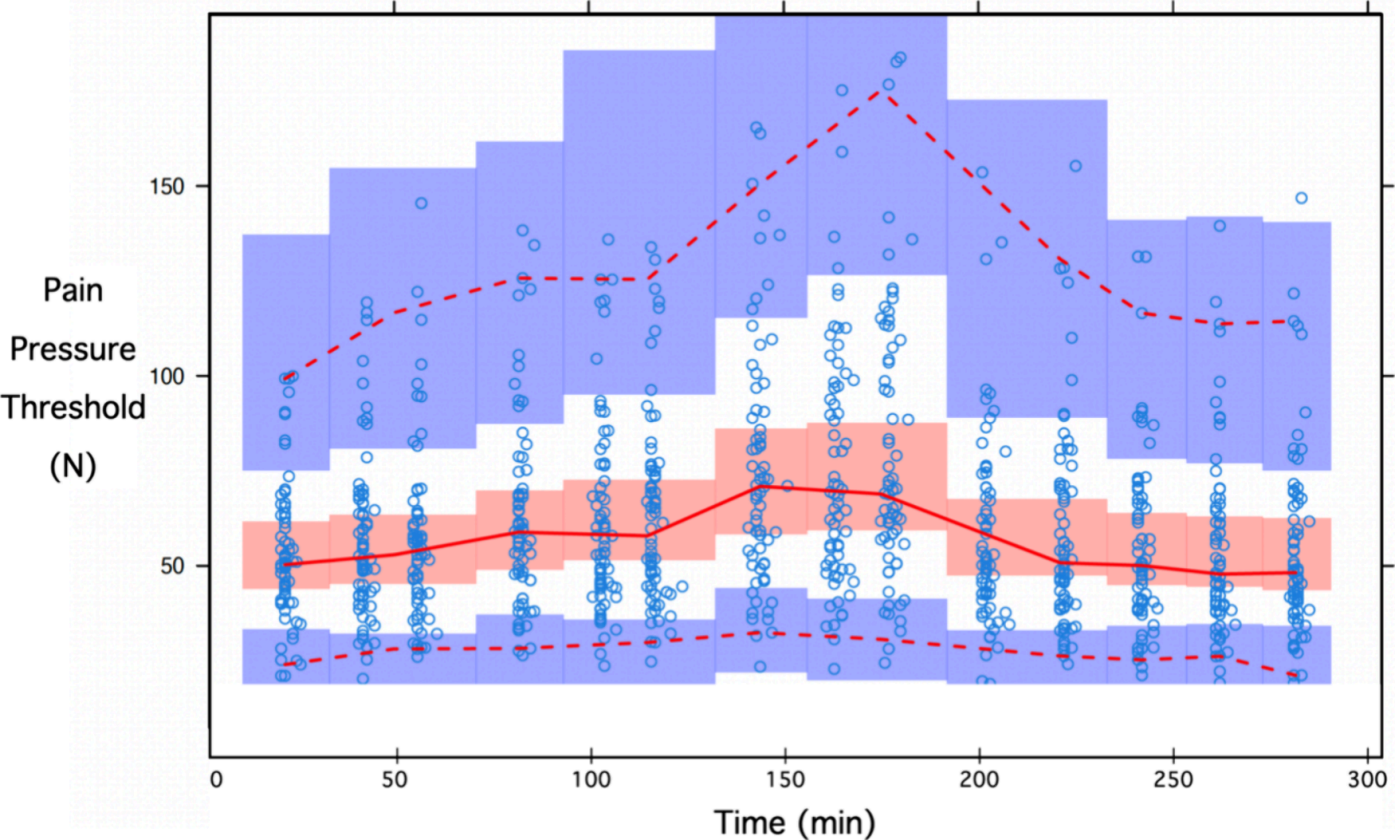
Visual predictive checks for the pressure pain threshold (open circles are the measured data observed during all four test conditions). The red line represents the 50th percentile of the observed data with the 95% confidence interval of simulated data (light red bars), and the upper and lower red broken lines are the 5th and 95th percentiles of the observed data with the 95% confidence intervals of simulated data (blue bars).

The results of the pharmacodynamic modeling analyses (final model, as given in Eqn. 8) are collected in Table 1 and Figures 2, 3 and 4. The individual pain pressure threshold data and corresponding data fits for the 4 conditions esketamine/placebo, esketamine/SNP, racemic-ketamine-placebo and racemic-ketamine/SNP, together with their population predicted pharmacodynamic outcomes (red lines) are given in Figure 2. Diagnostic of goodness-of-fit plots (observed vs individual predicted; observed vs population predicted; individual weighted residual vs time; normalized prediction discrepancy error vs time; and conditioned weighted residuals vs population predicted) are given in Figure 3 for the esketamine (panels A-E) and racemic ketamine experiments (panels F-J) with different symbols for data obtained without and with SNP. We present the esketamine and racemic ketamine data in distinct panels to allow a more precise review of the data, although the data were analyzed in a single model. The concentration-effect data for the 4 conditions are given in Figure 4. It shows the normalized effect, based on the Bayesian estimates of the baseline values, of the raw data, median measured data and median predicted data population fit versus median plasma concentrations of esketamine and racemic ketamine without and with SNP administration. The predicted data fits are in close agreement with the measured median data. The visual predictive check is given in Figure 5 for the complete data set analyzed with the final model (eq 9). Inspection of Figures 2–5 confirms the appropriateness of the pharmacodynamic model, although panels B and G (measured versus population predicted) indicate the large baseline variability in the data.

**Table 1:** Effect of Sodium Nitroprusside on S- and R-Ketamine and S- and R-Norketamine induced Changes in Pain Pressure Threshold and Influence^a^

	Value ± SEE (%RSE)	ω^2^ ± SEE (%CV)	ν^2^ ± SEE (%CV)
Baseline value (*N*)	48.9 ± 4.0 (8)	0.102 ± 0.037 (32)	0.024 ± 0.017 (15)
C_100,S_ ketamine (nmol/mL)	0.47 ± 0.12 (25)	-	-
C_100,S_ norketamine (nmol/mL)	0.25 ± 0.08 (31)	0.113 ± 0.059 (34)	0.152 ± 0.101 (40)
Factor SNP effect on S-ketamine and norketamine potency: λ_A_	1 FIX	-	-
Factor S- vs R-ketamine and norketamine effect on potency: λ_B_	0.52 ± 0.27 (51)		
Factor SNP effect on R-ketamine and norketamine potency: λ_C_	0 FIX	-	-
γ	2.4 ± 0.9 (37)	-	0.207 ± 0.118 (47)
*t*_1/2_*k*_e0_ (min)	13.2 ± 4.1 (31)	-	0.655 ± 0.178 (96)
σ	42.7 ± 7.9 (19)		

a SEE is the standard error of the estimate; RSE is the relative standard error; ω^2^ is interindividual variability; ν^2^ is interoccasion variability; CV is the coefficient of variation for interindividual or interoccasion variability, calculated as √[exp(ω^2^) – 1] × 100; C_100,s_ ketamine is the S-ketamine effect-site concentration causing a 100% increase in pain threshold value; C_100,S_ norketamine the S-norketamine effect-site concentration causing a 100% increase in ketamine C_100,S._ λ_A_ is a factor that determines the change of S-ketamine or S-norketamine antinociceptive analgesic potencies in the SNP group relative to the placebo group, λ_B_ is a factor that determines the change of R-ketamine and R-norketamine potencies relative to S-ketamine or S-norketamine in the placebo group; and λ_C_ is a factor that determines the change of R-ketamine or R-norketamine potencies in the SNP group relative to the placebo group; γ is a shape parameters; *t*_1/2_*k*_e0_ the blood-effect-site equilibration half-life; σ^2^ the residual error variance; – parameter not included in the model; FIX parameter fixed to a specific value (0 or 1, as indicated).

**Figure 6:**
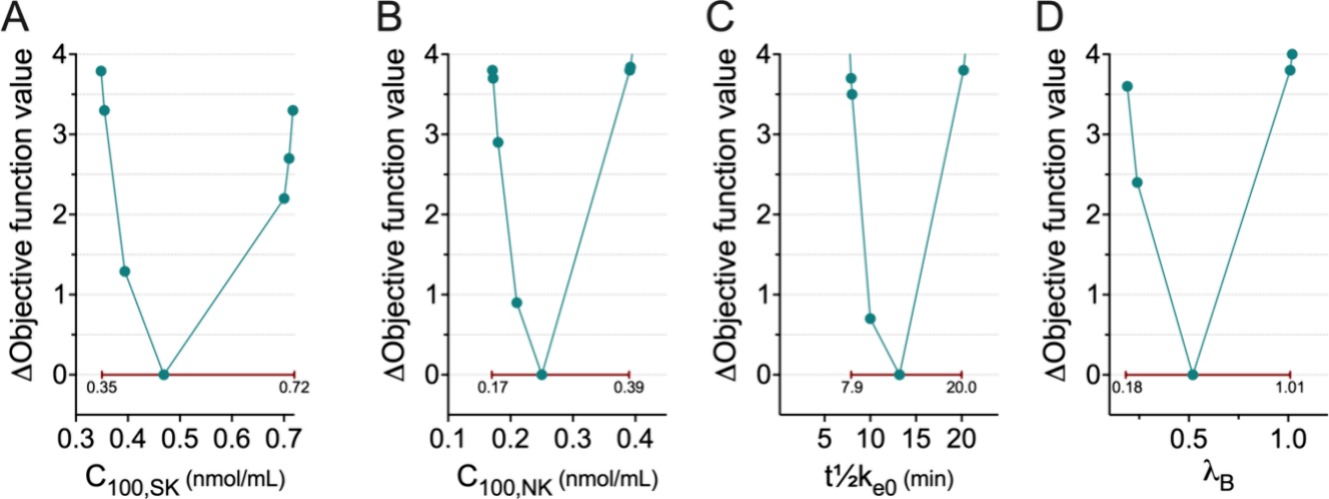
Log likelihood profiles for model parameters C_100,SK_ (A), C_100,NK_ (B), *t*_1/2_*k*_e0_ (C) and λ_B_. The red lines are the 95% confidence intervals. C_100,SK_ is the estimated S-ketamine effect-site concentrations needed to increase the pain pressure threshold by 100% relative to baseline; C_100,SN_ is the S-norketamine effect-site concentration causing a 100% increase in the S-ketamine C_100_ value; *t*_1/2_*k*_e0_ is blood effect-site equilibration half-life; λ_B_ is a factor that determines the change in C_100_ from S-ketamine and S-norketamine to R-ketamine or R-norketamine, respectively, in the placebo treatment group.

In order to assess the difference between ketamine enantiomers and SNP effect, we introduced parameter λ, which indicates whether a difference in analgesic potency is present between isomers and treatments; λ_A_ is a factor that determines the change of S-ketamine or S-norketamine analgesic potencies in the SNP group relative to the placebo group, λ_B_ is a factor that determines the change of R-ketamine and R-norketamine potencies relative to S-ketamine or S-norketamine in the placebo group; and λ_C_ is a factor that determines the change of R-ketamine or R-norketamine potencies in the SNP group relative to the placebo group. The analgesic potency is defined by parameter C_100_ or the ketamine (S or R) concentration causing a 100% increase in PTT from baseline, or the norketamine (S or R) concentration causing a 100% increase in ketamine C_100_. The reduction in ketamine potency (i.e., the increase in C_100_ value) is probably due to displacement of ketamine by norketamine, a metabolite with much less potency than ketamine; in our analysis the metabolite was without intrinsic activity. The final model (eq 8) with all λ’s freely estimable had an objective function value (OFV) of 5093 points. The values of the factors were λ_A_ = 0.98 ± 0.21, λ_B_ = 0.59 ± 0.51 and λ_C_ = 0.06 ± 0.41. λ_C_ was significantly different from 1 (OFV 5098), and since it was close to zero it was fixed to 0. Similarly, λ_A_ was fixed to 1. With fixed values for λ_A_ and λ_C_, λ_B_ was estimated as 0.52 ± 0.27 (95% CI 0.18–1.01; Figure 6). The resultant OFV of 5094 indicates that λ_A_ and λ_C_ were indeed not significantly different from 1 and 0, respectively.

Removing the norketamine effect from the model increased the OFV by about 200 points, and caused a significant misfit of the data. Additionally, if we did not incorporate norketamine in the final model, the value of *t*_1/2_*k*_e0_ became zero, a value to we did not consider realistic. The modeling results were such that the S-norketamine concentration dependent antagonism of ketamine doubled S-ketamine C_100_ at an effect-site concentration of 0.25 ± 0.08 nmol/mL. Such concentrations are observed after about 2 h of S-ketamine infusion and resulted in a lower analgesic response than anticipated (i.e., without an antagonistic S-norketamine effect), particularly after discontinuing the S-ketamine infusion. We earlier showed similar results for the dissociative S-ketamine effects and S-ketamine induced changes in cardiac output.^5,18^ Animal data indicate that norketamine is an NMDA antagonist with analgesic properties, albeit with much less potency than ketamine.^19,20^ This suggests that in our study, at later time points, norketamine effectively displaced ketamine from the NMDA receptor and since it has a lesser potency, caused a reduction in pain relief.

Note that the model we used (see Methods and Materials Section below) is based on receptor kinetics with the interaction of one full agonist (ketamine) and an antagonist with the latter being defined as a partial agonist with zero intrinsic activity (norketamine). With intrinsic activity α between 0 and 1, we have
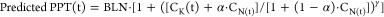
1However, fitting this model resulted in a minimum objective value when α = 0, which gives eq 9.

Since we estimated that λ_B_ = 0.52, the R-ketamine (C_100,RK_) and R-norketamine (C_100,RN_) potencies were half of that of S-ketamine (C_100,SK_ = 0.47 ± 0.12 nmol/mL, median ± SEE) and S-norketamine (C_100,SN_ = 0.25 ± 0.08 nmol/mL; Table 1). As derived from likelihood profiling, the 95% confidence intervals (CI) were for C_100,SK_ 0.35–0.72 nmol/mL and C_100,SN_ 0.17–0.39 nmol/mL (Figure 6). Given the observation that λ_A_ = 1, the S-ketamine and S-norketamine potencies were unaffected by SNP treatment, while the observation that λ_C_ = 0, indicates that the contribution of R-ketamine in the SNP groups was significantly different from its contribution in the placebo group. In fact, the very low λ_C_ value indicates the abolishment of R-ketamine contribution to the analgesic responses during SNP treatment.

The blood effect-site equilibration half-life (*t*_1/2_*k*_e0_) was estimated to 13.2 ± 4.1 min with 95% CI 7.9–20 min. This agrees with earlier observations,^16,21^ although other values are reported as well. These differences may be attributed to differences in infusion paradigm, sampling scheme, and species. For example, in rats both ketamine and norketamine measured brain concentrations peaked within 1 min of administration.^22^ While this does not directly correspond to parameter *t*_1/2_*k*_e0_, which reflexes the hysteresis between plasma drug concentration and effect, it does indicate that at least in rats the value of *t*_1/2_*k*_e0_ is probably shorter than in our current study.

R-ketamine is not readily available for human use, hence, we used PKPD modeling to get an indication of the potency of R-ketamine. With a 50% lesser analgesic R-ketamine potency compared to S-ketamine, the potency of racemic ketamine is reduced by about 30% to 0.62 ± 0.19 nmol/mL compared to esketamine. There is evidence that R-ketamine is a potent antidepressant.^23,24^ This was recently echoed by the observation in a single patient who experienced remission of major depression following administration of racemic-ketamine but not esketamine.^25^ Still, human studies show a much greater contribution of S-ketamine (≫ 50%) to a variety of end points, including pain relief, cardiac output, blood pressure effects and EEG suppression, when compared to either R-ketamine or racemic-ketamine.^5,18,26,27^ For example, the anesthetic potency of S-ketamine is about 55% greater than that of R-ketamine and 60% greater than that of the racemate.^27^ Similarly, an increase in mean arterial pressure was greater after administration of S-ketamine (total intravenous dose given 1 mg/kg) than after the racemic mixture (2 mg/kg).^26^ In the placebo groups we observed that the R-ketamine analgesic potency as defined by parameter C_100_ was about half of that of S-ketamine, causing a 30% difference in potency between esketamine and racemic ketamine. We relate these stereoselective effects to differences in NMDA receptor effects with S-ketamine being twice as potent as R-ketamine in producing a voltage-dependent and use-dependent blockade of the receptor, possibly related to engagement of different NMDA receptor subunits.^17,28^ The difference in stereoselective effects on different end points (*e.g*. pain relief versus mood improvement) mirrors the complexity of ketamine’s pharmacology.

SNP is a potent nitric oxide donor.^5^ Ample animal data show that neuronal nitric oxide depletion following NMDA receptor blockade plays an important mechanistic role in the generation of ketamine-induced dissociative symptoms by blocking the NMDA receptor, and subsequently inhibiting the glutamate-cGMP pathway.^5−9^ Upon stimulation of the NMDA receptor by glutamate, this pathway results in NO-dependent neuroplasticity, neurotrophic effects and neuroprotection. Our current findings indicate that neuronal NO suppletion affects the analgesic response. Particularly, modeling showed that R-ketamine analgesic response were sensitive to NO exposure. Model parameter λ_C_ (initial estimation 0.06, later fixed to 0) indicates that NO virtually abolished the R-ketamine contribution to effect. Similar observations were made previously for the effect of NO on racemic-ketamine but not esketamine-induced dissociative symptoms. Racemic ketamine-induced dissociation is reduced during SNP infusion by 20–40%. The question remains whether our current observation is due to a direct NMDA-independent NO effect,^29,30^ an NMDA-dependent effect on R-ketamine activated pathways or is related to the reduction in dissociative symptoms.^5^ A direct NO effect that is independent of the NMDA receptor seems unlikely as SNP did not diminish the esketamine (i.e., the S-ketamine) analgesic effects. Our currents study design does not allow distinction between a direct NO effect that and occurs downstream of receptor blockade from an effect related to a specific mechanistic effect of reduced dissociative symptoms. It has been argued that the conscious perception of psychedelic dissociative symptoms from ketamine or other psychedelic or psychoplastic drugs are necessary for their therapeutic or desirable effects.^31,32^ For example, psilocybin subjective effects mediate the magnitude of addiction therapy: the greater the subjective experience, the greater the desired change in addiction behavior.^33^ We earlier showed in our study population that there is a connectivity between esketamine induced dissociation and analgesia.^16^ Both end points were generated with similar potency and dynamics and consequently, we inferred a neurobiological mechanistic link between dissociation and modulation of pain pathways. Our current study suggests that the up to 40% reduction in dissociative effects that we observed in the current population during SNP and racemic ketamine infusion (see ref (5)) might be relevant to the reduced analgesic effects. Still, we cannot exclude that we are facing a simultaneous effect of NO on R-ketamine associated dissociation and an effect of NO on nociceptive pathways, but without mechanistic link. Further studies are needed to explore this matter.

Some study limitations need to be discussed. (i) Our current secondary analysis was not powered to study an SNP effect on nociception. Hence, we consider this a hypothesis generating and exploratory study. (ii) We studied the effect of two drug combinations (S- and RS-ketamine/placebo versus S- and RS-ketamine/SNP) and possibly complex drug–drug interactions were not captured in our analysis. An example of an interaction is the observation that SNP causes a 9% increase in ketamine clearance and 22% increase in intercompartmental clearances,^34^ possibly due to an increase in ketamine hepatic extraction from blood related to a higher liver perfusion during SNP treatment. No such effects were detectable for the ketamine metabolites. Our current analysis, however, does take these differences in plasma concentrations that occurred because of the pharmacokinetic effects of SNP into account. (iii) The analgesic assays we used may have limited sensitivity to the effects of nitric oxide during esketamine infusion. Possibly studying the effect of the ketamine/SNP treatment on neuropathic pain or pain conditions with signs of central sensitization may elucidate interactive effects that we did not detect in our study in healthy volunteers with standardized experimental nociceptive assays. (iv) We performed complex modeling. We are confident that our model adequately described the data with parameter estimates that are in line with previous studies. However, due to the complexity of the protocol and the modeling that we performed, we may have overinterpreted the data. Still, the outcome of our analysis is in line with earlier observations of the ability of NO to modulate ketamine effects. This gives us further confidence that our exploratory approach gave reliable results. (v) Apart from its effects at the NMDA receptor, ketamine has agonist activity at the μ-opioid receptor, as observed for esketamine in exon 2 μ-opioid receptor knockout mice.^35^ In our analysis we did not consider an effect of ketamine at the opioid receptor system. Since we do not assume that the opioid effect is dependent on SNP, it may be that the remnant analgesic effect observed during SNP administration is related to an interaction with the μ-opioid receptor. This would then suggest that R-ketamine is devoid of activity at the μ-opioid receptor, while S-ketamine’s analgesic effect is predominantly related to its agonistic activity at the opioid receptor. Further studies are needed to address this issue in humans.

In conclusion, our comprehensive analysis revealed insights into the mechanisms underlying the analgesic properties of ketamine. We observed that R-ketamine activation of analgesic pathways is dependent on NO-donor SNP. In contrast, SNP did not exert any discernible influence on the analgesic effects of S-ketamine. These contrasting findings imply that a non-NMDA receptor-mediated direct pronociceptive effect induced by SNP is unlikely responsible for modulating R-ketamine analgesia. We therefore conclude that SNP interacts with R- but not S-ketamine nociceptive pathways downstream from the NMDA receptor, conceivably similar to its effects on R-ketamine activated dissociation pathways. A possible role of the reduced dissociative symptoms in the R-ketamine effect requires further study.^36^

## Methods and Materials

### Ethics, Registration, and Subjects

This study is a preplanned secondary analysis of a data set aimed to study the influence of SNP on the dissociative effects of esketamine and racemic ketamine (i.e., the primary analysis).^5^ Additional secondary analyses were the development of a population pharmacokinetic model of S- and R-ketamine and their metabolites,^34^ a pharmacodynamic model of the influence of S- and R-ketamine on cardiac output,^18^ and an integrated pharmacodynamic model of S and R-ketamine and metabolite norketamine on the connection of analgesic and dissociative effects.^16^ Here we present a pharmacodynamic model on the influence of SNP on R- and S-ketamine and R- and S-norketamine induced analgesia.

The study had a double-blind, crossover, 4-arm design and was performed at a single center, Leiden University Medical Center, after approval of the protocol by the local ethics committee (Commissie Medische Ethiek, Leiden, The Netherlands) and the Central Committee on Research involving Human Subjects (Centrale Commissie Mensgebonden Onderzoek, the Hague, The Netherlands). All subjects gave written informed consent before participation in the study. The study was registered in the Dutch trial register registered at the register of the Dutch Competent authority on August 8, 2015.^37^ The study was conducted in 17 healthy male volunteers with mean age ± SD of 23 ± 3 years, and with a body mass index of 24 ± 2 kg/m^2^. Specific in- and exclusion criteria were published elsewhere.^5^ Subjects were recruited through flyers on the university campus.

### Drug Infusion

The participants were tested on four occasions, receiving the esketamine, just containing the S-enantiomer (Ketanest-S, Eurocept BV, The Netherlands) combined with SNP or placebo (normal saline), or racemic-ketamine, containing both S- and R-enantiomers (Ketalar, Pfizer, Germany), combined with SNP or placebo. The subjects were randomized with respect to ketamine formulation and the infusion of SNP versus placebo using a computer-generated randomization list. All participants completed all 4 treatment sessions. The data for the current *post hoc* analysis were collected on four distinct days in which racemic or esketamine was administered with either SNP or placebo on separate days.

On each study day, we inserted two intravenous infusion lines and one arterial line, for drug administration (ketamine and SNP were infused in contralateral arms) and arterial drug sampling (the arterial line was placed in left or right radial artery, but always opposite from the ketamine infusion arm). Racemic- and esketamine were continuously administered for 3 h at an escalating infusion rate. Racemic-ketamine: first hour 0.28 mg/kg, second hour 0.57 mg/kg and third hour 1.14 mg/kg, with a total dose of 2 mg/kg; Esketamine: first hour 0.14 mg/kg, second hour 0.28 mg/kg and third hour 0.57 mg/kg, with a total dose of 1 mg/kg. On two study days SNP (Apotheek Haagse Ziekenhuizen, the Hague, The Netherlands) administration was started 1 h before the ketamine infusion and continued until the end of the ketamine infusion; on the other study days placebo (0.9% NaCl) was given instead of SNP. The infusion of placebo or SNP 0.5 mg/kg per min started 1 h prior to ketamine dosing and continued until the end of ketamine administration. At this dose SNP was without hemodynamic effects in our healthy volunteers.^18^

### Measurements: Plasma Concentrations

Arterial blood samples were obtained at regular intervals (t = 2, 6, 30, 59, 62, 66, 100, 119, 122, 126, 150, 179, 182, 186, 195, 210, and 300 min after the start of ketamine infusion) for measurement of the concentrations of R- and S-ketamine, R- and S-norketamine and total hydroxynorketamine. The pharmacokinetic data were analyzed using an enantioselective assay for ketamine and norketamine, but not for hydroxynorketamine, by high-performance liquid chromatography-tandem mass spectrometry after solid-phase extraction. The analyses were performed in the Anesthesiology laboratory of at the Washington University School of Medicine (St. Louis, MO, USA). For ketamine and norketamine the lower and upper limits of quantitation were 2.5 and 250 ng/mL, respectively, and for hydroxynorketamine 5 and 500 ng/mL.^34^

### Measurements: Analgesia

We applied a pressure pain test to determine the effect of ketamine on pain relief. Prior to any ketamine infusion, baseline analgesic data were obtained after which the tests were repeated at regular intervals until 2 h after the racemic ketamine infusion. During ketamine infusion 3 tests were performed in each hour, while 5 tests were performed in the 2 h following infusion. To induce pressure pain, a 1 cm^2^ circular flat metal surface was applied at increasing pressure on the skin between the thumb and index finger while the hand was position on a flat solid surface. We used the FP 100 N Algometer (FDN 100, Wagner Instruments Inc., USA), with a force capacity ± accuracy of 100 ± 2 N. When the applied pressure was perceived as painful, the pressure was released and the force noted (i.e., the pressure pain threshold).

### Data Analysis

For the current analysis, no sample size estimation was conducted, as this was a *post hoc* analysis of the data. The original study was powered to detect a 20 ± 20% (mean ± SD) reduction of ketamine-induced dissociation by SNP;^5^ 17 subjects were needed to attain at least 80% power at an α-level of 0.05. We included 20 subjects to consider for the withdrawal of subjects or loss of data for any reason. Since this is an exploratory study, we did not infer any *a priori* significance level. We do report objective function values (OFV) for the various steps taken in the modeling process.

### Pharmacokinetic-Pharmacodynamic Model Analysis

The pharmacokinetic-pharmacodynamic analysis focused on an S- and R-ketamine effect on pain pressure threshold and was performed in NONMEM version 7.5.1 (ICON Development Solution, USA). The pharmacokinetic model consisted of two compartments for ketamine, norketamine and hydroxynorketamine each, and one compartment for one additional ketamine metabolite, dehydronorketamine. Two delay compartments were included between the ketamine central compartment and the norketamine central compartments. We included R-ketamine, S-ketamine, R-norketamine and S-norketamine in our analysis. The results of the pharmacokinetic analysis were published before.^40^ Empirical Bayesian estimates were derived from the pharmacokinetic analysis and served as input to the pharmacodynamic model. A possible delay between plasma concentration and effect was modeled by assuming an effect-compartment that equilibrated with the central pharmacokinetic compartment with half-life *t*_1/2_*k*_e0_ (= ln(2)/*k*_e0_).

Pain pressure responses were analyzed as follows:^16^
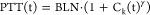
2and
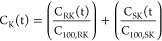
3where PPT(t) is the pain pressure threshold or the amount of pressure (unit Newton) applied at which the participant first reported pain at time *t*, BLN is the estimated pressure pain threshold at baseline, C_SK_(t) and C_RK_(t) are the effect-site concentration of S- and R-ketamine (units nmol/mL) at time *t*, C_100,SK_ and C_100,RK_ are the estimated S- and R-ketamine effect-site concentrations needed to increase the pain pressure threshold by 100% relative to baseline, and γ a shape factor.

From these initial analyses we observed that the effect of R-ketamine deserved further tuning (see below) and that the potencies of ketamine and norketamine were highly correlated. Additionally, we observed that responses declined somewhat at high ketamine and norketamine concentrations that this caused a small misfit when just including ketamine as input to the pharmacodynamic model. We therefore included R- and S-norketamine as an additional input to the model. We assumed, according to a receptor kinetic approach, that norketamine could displace ketamine from the *N*-methyl-d-aspartate (NMDA) receptor. A consequence is that norketamine, given its lower potency than taht of ketamine, has a seemingly nociceptive rather than an analgesic effect (see also eq 1, where norketamine is defined a partial agonist without intrinsic activity).

We define C_N_ as follows:^16^
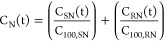
4where C_SN_(t) and C_RN_(T) are the S- or R-norketamine plasma concentration in nmol/mL and C_100,SN_ and C_100,RN_ the S- or R-norketamine effect-site concentration causing a 100% increase in the S- or R-ketamine C_100_ values, respectively.

To determine possible differences in effect of R-ketamine versus S-ketamine, and R-norketamine versus S-norketamine, on their respective potencies (C_100_) and to determine the effect of SNP versus placebo, we constructed the following models:

Placebo treatment, S-ketamine versus R-ketamine:
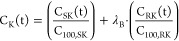
5
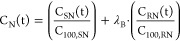
6

SNP versus placebo treatment:
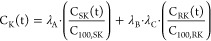
7
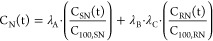
8where λ_A_ is a factor that determines the change of C_100_ of S-ketamine or S-norketamine in the SNP group relative to the placebo group, λ_B_ is a factor that determines the change of C_100_ of R-ketamine and R-norketamine relative to S-ketamine or S-norketamine in the placebo group; and λ_C_ is a factor that determines the change of C_100_ of R-ketamine or R-norketamine in the SNP group relative to the placebo group. If λ_A_ and λ_C_ equal to 1, there is no differences in C_100_ between placebo and SNP groups for both the parent and metabolite. If either λ_A_ or λ_C_ equal to zero, there is no contribution of the ketamine and norketamine enantiomers in the SNP group (λ_A_ = 0, no contribution of S-ketamine and S-norketamine; λ_C_ = 0, no contribution of R-ketamine and R-norketamine). If λ_B_ = 1, the potencies of S- and R-ketamine (and S- and R-norketamine) are similar in the placebo groups.

The predicted value of the pain pressure threshold is then given by eq 1 with α = 0:
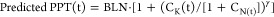
9This model has additive effects for the S- and R-ketamine enantiomers but with possible different potencies. The pharmacodynamic model further included random effects to consider interindividual variability (ω^2^) and interoccasion variability (ν^2^): θ_i_ = θ × exp(η_i_), where θ_i_ is the parameter for individual i, θ the population parameter and η_i_ is the random difference between the population and individual parameters, where its variance is the sum of ν^2^ and ω^2^.

In addition to the $COV step in NONMEM to determine the standard error of the (parameter) estimate, Perl-speaks-NONMEM (PsN) log likelihood profiling (llp) utility was used to determine the 95% confidence intervals for parameters S- and/or R-ketamine C_100_, S- and/or R-norketamine C_100_ and *t*_1/2_*k*_e0_. Values are median ± standard error of the estimate (SEE).

## Abbreviations

PD: pharmacodynamics
NK: norketamine
NO: nitric oxide
OFV: objective function value
PK: pharmacokinetics
PPT: pain pressure threshold
RK: R-ketamine
SK: S-ketamine
SNP: sodium nitroprusside
